# Using provider-focused education toolkits can aid enhanced recovery programs to further reduce patient exposure to opioids

**DOI:** 10.1186/s13741-020-00153-5

**Published:** 2020-07-09

**Authors:** Ankit Sarin, Elizabeth Lancaster, Lee-lynn Chen, Sima Porten, Lee-may Chen, Jeanette Lager, Elizabeth Wick

**Affiliations:** 1 Department of Surgery, University of California San Francisco, 550 16th Street, 6th Floor, San Francisco, CA 94158 USA; 2 Department of Anesthesia & Perioperative Medicine, University of California San Francisco, 505 Parnassus Ave. M917, San Francisco, CA 94143-0624 USA; 3 Department of Urology, University of California San Francisco, 1825 4th Street, Fourth Floor, San Francisco, CA 94158 USA; 4 Department of Obstetrics, Gynecology & Reproductive Sciences, University of California San Francisco, 550 16th Street, 7th Floor, San Francisco, CA 94158 USA

**Keywords:** Opioid reduction, Surgical pathways, ERAS, Postoperative, Provider education

## Abstract

**Background:**

Evidence-based perioperative analgesia is an important tactic for reducing patient exposure to opioids in the perioperative period and potentially preventing new persistent opioid use.

**Study design:**

We assessed the impact of a multifaceted optimal analgesia program implemented in the setting of a mature surgical pathway program at an academic medical center. Using existing multidisciplinary workgroups established for continuous process improvement in three surgical pathway areas ((colorectal, gynecology, and urologic oncology (cystectomy)), we developed an educational toolkit focused on implementation strategies for multimodal analgesia and non-pharmacologic approaches for managing pain with the goal of reducing opioid exposure in hospitalized patients. We analyzed prospectively collected data from pathway patients before dissemination of the toolkit (July 2016–June 2017; *n* = 869) and after (July 2017–June 2018; *n* = 838). We evaluated the association between program implementation and use of oral morphine equivalents (OME), average pain scores, time to first ambulation after surgery, urinary catheter duration, time to solid food after surgery, length of stay, discharge opioid prescriptions, and readmission.

**Results:**

Multivariate regression demonstrated that the program was associated with significant decreases in intraoperative OME (14.5 ± 2.4 mEQ (milliequivalents) reduction; *p* < 0.0001), day before discharge OME (18 ± 6.5 mEQ reduction; *p* < 0.005), day of discharge OME (9.6 ± 3.28 mEQ reduction; *p* < 0.003), and discharge prescription OME (156 ± 22 mEq reduction; *p* < 0.001). Reduction in OME was associated with earlier resumption of solid food (0.58 ± 0.15 days reduction; *p* < 0.0002).

**Conclusion:**

Our multifaceted optimal analgesia program to manage perioperative pain in the hospital was effective and further improved analgesia in the setting of a mature enhanced recovery program.

## Introduction

The US opioid epidemic is a major public health crisis, costing the healthcare system over $1 trillion since 2001 and projected to exceed another $500 billion over the next 3 years (Altarum, 2019). New persistent opioid use following surgery is of such importance it is now considered a postoperative complication, much like venous thromboembolism or surgical site infection (Waljee et al., 2017). An estimated 5–19% of opioid-naive patients continue to use opioids after the immediate postoperative period, following both major and minor procedures (Alam et al., 2012; Clarke et al., 2014; Johnson et al., 2016; Schoenfeld et al., 2017; Shah et al., 2017). Furthermore, one in four opioid prescriptions filled in the USA is for acute pain following procedural care, and surgeons are considered gatekeepers to opioid prescriptions. Substantial efforts have been made on local and national levels to reduce opioid exposure for patients undergoing surgery (Brummett et al., 2017; Clarke et al., 2014; Cron et al., 2018; Eid et al., 2018; Hanson et al., 2018; Hill et al., 2017; Howard et al., 2018; Overton et al., 2018; Shah et al., 2017; Thiels et al., 2017; Waljee et al., 2017).

Enhanced recovery was first described in Europe in the 1990s as a perioperative program aimed at using evidence-based processes of care to maintain pre-operative organ function and reduce the profound stress response following surgery. Over the past decade, adoption of enhanced recovery has exploded in the USA, where surgical pathways or enhanced recovery pathways (ERPs), in addition to reducing the stress of surgery, have been an important mechanism to decrease variability in practice, reduce morbidity, shorten postoperative length of stay (LOS), and improve the value of surgical care. Results from published reports (Geltzeiler et al., 2014; Gillissen et al., 2013; Huibers et al., 2012; Lohsiriwat, 2014; Teeuwen et al., 2010) and systematic reviews (Bagnall et al., 2014; Eskicioglu et al., 2009; Rawlinson et al., 2011; Wind et al., 2006) have been encouraging, and we have shown the benefit of this approach in patients who have had colorectal surgery (Sarin et al., 2016) both immediately and at 6-month follow-up (Deiss et al., 2018), as well as in gynecologic oncology patients (Chapman et al., 2016). As the opioid crisis evolved, it has become clear that even with brief exposure to opioids after surgery, one in six patients are at risk for persistent opioid use (Brummett et al., 2017; Sun et al., 2016). Although ERP guidelines include multimodal analgesia as key components, recommendations regarding opioid analgesia and non-pharmacologic strategies to manage pain are not included. Therefore, we hypothesized that a focused effort on appropriate opioid use in the setting of ERP-based multimodal analgesia had the potential to further reduce patient opioid exposure. We therefore sought to demonstrate the effectiveness of an education-focused approach to opioid reduction in mature surgical pathways. We hypothesized that our existing surgical pathway structure could be harnessed to emphasize optimal analgesia and further reduce opioid exposure during the perioperative period.

## Methods

### Study design

This was a retrospective analysis of prospectively collected data before (July 2016 to June 2017) and after (July 2017 to June 2018) the implementation of an opioid-reducing educational toolkit to evaluate its effect. The Departments of Surgery, Anesthesiology, Gynecology, Urology, and Nursing were the target audience, and the effort was layered on a surgical pathway program that was started for colorectal surgery in 2013, gynecologic oncology in 2014, benign gynecology in 2015, and urologic oncology in 2016, as previously described (Chapman et al., 2016; Sarin et al., 2016) (Supplementary Material 1). This study was approved by the Institutional Review Board at the University of California—San Francisco (UCSF): study number 18-26677.

### Educational intervention

The education strategy focused on the risk of new persistent opioid use in surgical patients and potential strategies to reduce exposure to opioids and decrease that risk. The strategy, including the toolkit, was designed specifically to address the different phases of surgical care and provider groups, as follows. The immediate perioperative (non-operating room) group consisted of nurses and anesthesia providers in the holding area and recovery room. The intraoperative group comprised of surgeons and anesthesia providers. The postoperative group consisted of surgeons, surgical residents, pain management providers, advanced practice providers, inpatient unit nurses, and the anesthesia-led pain service. The intervention was deployed with each group in July 2018. The sessions were interactive and led by a designated surgeon and anesthesiologist.

### Educational toolkit

The multifaceted toolkit included the following components (Table 1):

1. A detailed intra-operative anesthesia protocol that included specific information about adjuncts (indications and dosing) including intravenous lidocaine, magnesium, and ketamine.
2. Protocol to discuss analgesia plan including adjuncts and intention for opioid-sparing analgesia during the universal protocol or time out at the beginning of the procedure and including a plan for non-steroidal anti-inflammatory administration during the debriefing at the end of the procedure.
3. Monthly didactic conference with surgeons, surgical residents, anesthesiologists, and anesthesia residents to review patient cases and best practices in perioperative analgesia.
4. In-service to inpatient unit nursing (three times a year) conducted by surgeon and anesthesiologist lead.
5. Partnership with unit-based “pain champion nurses” to reinforce messaging between sessions.
6. A nursing analgesia resource book (Supplementary Material 2).
7. Centralized location of all recommendations, pathways, and intraoperative protocols on websites (https://eras.ucsf.edu/ and https://anesthesia.ucsf.edu/clinical-resources-type/eras-pathway).

**Table 1:** Education toolkit

Perioperative nursing	Intraoperative providers	Postoperative providers
**Educational focus**		
Evaluation of surgical versus non-surgical (gas) pain	Minimizing intraoperative opioid use	Setting patient expectations regarding postoperative pain
Use of non-medical interventions as first line (heat packs, ice packs)	Advocating use of TAP blocks or local anesthesia	Avoiding escalating opioid use without discussing risk and benefit with patients
Recommendations regarding escalation of analgesic interventions with use of opioids last	Running epidurals intra-operatively when available	Recommendations regarding escalation of non-pharmacologic analgesia and pharmacologic analgesia
Advocating use of multimodal analgesia	Use of multimodal analgesia (magnesium, lidocaine if appropriate, Toradol)	Use of multimodal analgesia
**Educational strategy**		
Quarterly in-services	Written protocol	Monthly didactic conference
Standard booklet	Monthly didactic conference	Monthly orientation and handbook
Website	Website	Website

### Data collection

Our primary outcome of interest was oral morphine equivalents (OME) administered in the different phases of care. Secondary outcomes were average pain scores, time to first ambulation after surgery, urinary catheter duration, time to solid food after surgery, length of stay after surgery, discharge opioid prescribing, and readmission. All patient data were collected from an electronic medical records data warehouse. To measure opioid use, all opioids were converted to using a conversion table (Supplementary Material 3) based on published data (McPherson, 2014). The OME data was separated by phases of care as follows: intraoperative, 0–12 h postoperatively, 12–24 h postoperatively, 24–36 h postoperatively, the day before discharge, and the day of discharge. Discharge OME quantity was calculated using the sum of all opioid medications prescribed at discharge. Multimodal was defined as the patient being given two or more non-opioid pharmacologic analgesics for the first 48 h after surgery (Ban et al., 2019). The case mix index (CMI) is the average relative diagnosis-related group (DRG) weight of a hospital’s inpatient discharges and reflects the diversity, clinical complexity, and resource needs of all the patients in the hospital. A higher CMI indicates a more complex and resource-intensive case load (https://healthdata.gov/dataset/case-mix-index, n.d.).

### Statistical analysis

Continuous data is summarized as mean and standard deviation (SD) or median and interquartile range (IQR), and categorical data as proportions. Primary and secondary outcomes were first compared between the pre-implementation and post-implementation groups without adjusting for any confounding variable. Next, multivariate regression analysis was used to determine correlations between the outcomes and the two groups adjusting for age, gender, service line, ASA score, opioid use at the time of admission (including methadone), surgical approach (minimally invasive vs open), case mix index (CMI), epidural use, and multimodal analgesic use. The statistical significance for comparisons was set at a two-tailed alpha < 0.05. All analyses were performed using SAS version 9.4 (SAS Institute, Cary, NC, USA).

## Results

There were 869 patients in the pre-implementation group (July 2016 to June 2017) and 838 in the post-implementation group (July 2017 to June 2018) (Table 2). Age, sex, American Society of Anesthesia physical status, CMI, and surgical approach (open vs minimally invasive) were similar for both groups, but the percentage of robotic compared to laparoscopic cases was higher in the post-implementation group. The groups were generally balanced with regard to colorectal and gynecologic patients, but the post-implementation group had fewer cystectomy patients. The two groups did not differ in the number of patients on opioids or methadone at the time of surgery. Epidural use decreased post-implementation, but the use of multimodal analgesia was consistent before and after implementation

**Table 2:** Patient and surgery characteristics

	Pre-implementation, ***N*** = 869	Post-implementation, ***N*** = 838
**Demographics**		
Age, median (inter-quartile range)	54 years (43–66)	57 years(43–67)
Women	68.8%	69.9%
ASA rating 2–3	89%	93.6%
Ongoing opioid use	33.8%	33.2%
Methadone users	2.3%	2.4%
**Surgical service**		
Colorectal	50.1%	50.1%
Gynecology	42%	42.1%
Urology (cystectomy)	7.2%	6.6%
**Operative details**		
Laparoscopic	31.9%	29.9%
Robotic	15.3%	17.9%
Case mix index*, median (inter-quartile range)	1.95 (1.61–2.54)	1.96 (1.64–2.47)
**Anesthesia details**		
Epidural use	35.1%	27.7%
Use of 2 or more multimodal analgesics for the first 48 h	43.6%	44.2%

*The case mix index (CMI) is the average relative diagnosis-related group (DRG) weight of a hospital’s inpatient discharges, calculated by summing the Medicare Severity-Diagnosis Related Group (MS-DRG) weight for each discharge and dividing the total by the number of discharges. The CMI reflects the diversity, clinical complexity, and resource needs of all the patients in the hospital

### Unadjusted analyses

**Fig. 1:**
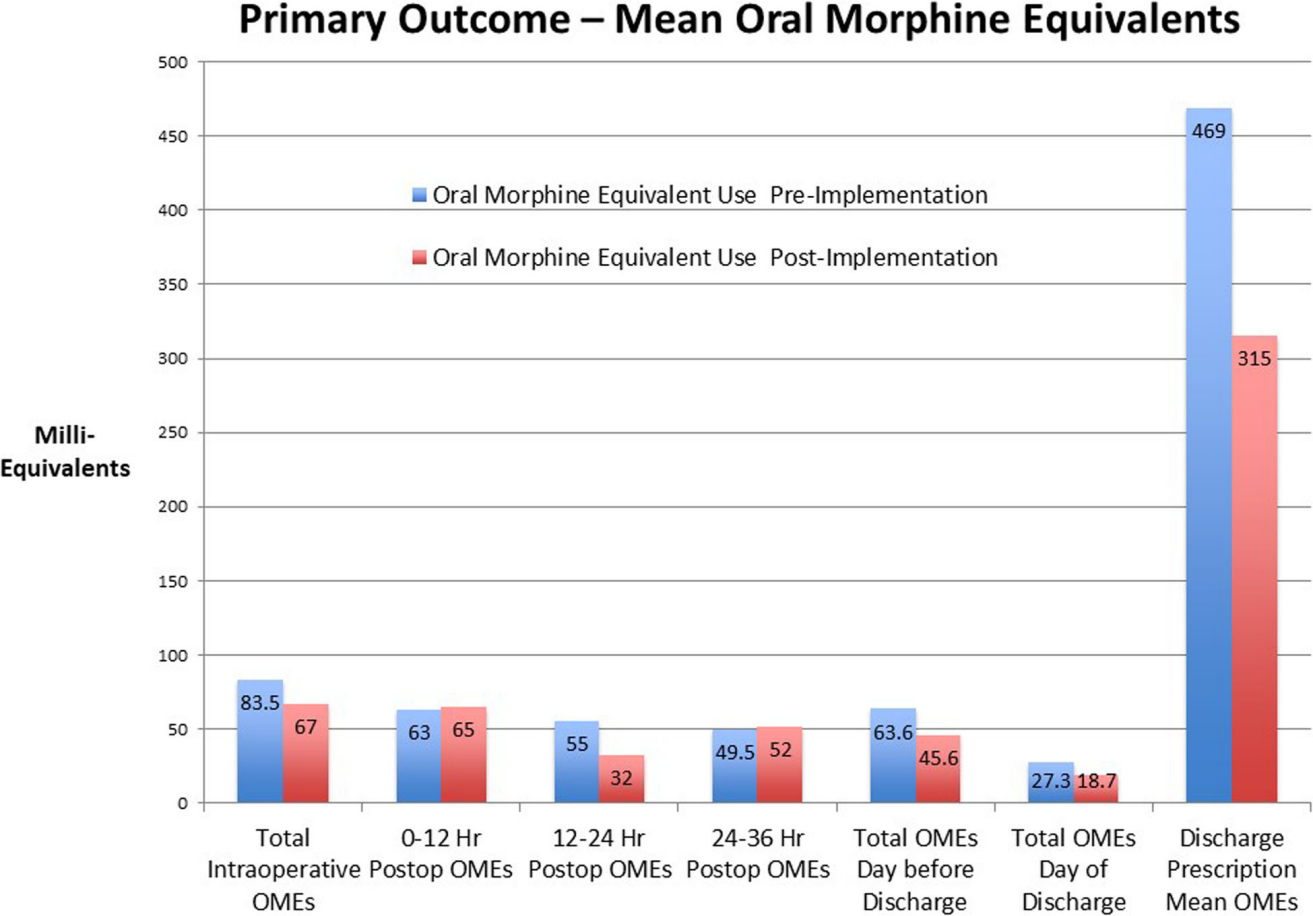
Mean oral morphine equivalents in milliequivalents in the different phases of care. Both pre-implementation and post-implementation average OMEs are shown without adjusting for any other variables

As shown in Fig. 1, mean OMEs decreased in the post-implementation group in the following phases of care: intraoperative, 12–24 h postoperatively, 24–36 h postoperatively, the day before discharge, day of discharge, and discharge prescriptions while they were slightly increased at the 0–12 h postoperative and the 24–36 h postoperative phases.

Table 3 shows that on unadjusted comparison, there was a decrease in post-procedure length of stay, 30 days readmission rates, and time to solid food. Average pain score on postoperative days (POD) 1 and 2 and time to first ambulation were slightly increased. Discharge prescription opioids were decreased. There was no change in duration of urinary catheter.

**Table 3:** Secondary outcome measures—unadjusted comparison (univariate)

	Pre-implementation, ***N*** = 869	Post-implementation, ***N*** = 838	***p*** value
**Recovery**			
Post-procedure LOS, days; mean (SD)	5.13 (5.02)	4.86 (5.3)	0.29
Time to first ambulation, hours; mean (SD)	15.50 (14.3)	16.21 (18.7)	0.41
Urinary catheter duration, hours; mean (SD)	44.16 (46.5)	44.77 (43.8)	0.80
Time to solid food, days; mean (SD)	2.63 (3)	1.95 (2)	< 0.0001
**Pain**			
Postop day 1, average pain score++; mean (SD)	2.6 (1.9)	3 (1.9)	0.0001
Postop day 2, average pain score; mean (SD)	2.6 (1.9)	3 (1.8)	0.0003
Discharge opioid, Rx quantity; OME mean (SD)	469 (528)	315 (433)	< 0.001
**Morbidity**			
Return to operating room within 30 days	3.11%	3.22%	0.89
Readmission within 30 days	17.26%	14.56%	0.13

*LOS* length of stay

^++^Pain scores are based on visual analog scale from 0 to 10

### Multivariate analyses

Multivariate regression analysis, adjusting for age, gender, service line, ASA score, opioid use at admission, surgical approach (minimally invasive vs open), CMI, epidural use, and multimodal analgesic use, showed that patients having surgery post-implementation had a significant decrease in OME exposure intra-operatively, on the day prior to discharge, and on the day of discharge but not in the first 24 h postoperatively (Table 4). Following implementation of the toolkit, there was a significant reduction in days to solid food and the quantity of opioids that patients were discharged on decreased significantly but there was no significant changes in post-procedure length of stay, post-procedure time to ambulation or urinary catheter duration, risk of return to operating room, or likelihood of readmission (Table 5). The average postoperative pain scores on a visual analog scale (POD 1 and 2) were modestly higher in the post-implementation period.

**Table 4:** Results of multivariate regression analysis—primary outcomes

	Estimate compared to pre-implementation year*	95% confidence limits	*p* value
**Intraoperative OME**	**− 15 milliequivalents**	**− 10.3 to − 19.9**	**< 0.0001**
0–12 h postop OME	5.1 milliequivalents	− 28 to 38.6	0.76
12–24 h postop OME	− 29.5 milliequivalents	− 62.6 to 3.5	0.08
**24-36 h postop OME**	**− 22.8 milliequivalents**	**− 43 to − 2.4**	**0.03**
**Total OME day before discharge**	**− 18 milliequivalents**	**− 5.7 to − 31**	**0.005**
**Total OME day of discharge**	**− 9.9 milliequivalents**	**− 16 to − 3.5**	**0.003**

*OME* oral morphine equivalents

*Covariate of interest was post-implementation year (compared with pre-implementation year). Models accounted for age, gender, service line, American Society of Anesthesiologist (ASA) rating, opioid use at the time of admission (including methadone), surgical approach (minimally invasive vs open), case mix index, epidural use, and multimodal analgesic

**Table 5:** Results of multivariate regression analysis—secondary outcomes

	Estimate compared to pre-implementation year*	95% confidence limits	*p* value
**Discharge opioid quantity, OME**	**− 156 milliequivalents**	**− 199 to − 112**	**< 0.001**
Post-procedure length of stay	− 0.24 days	− 0.7 to 0.3	0.3
**Postop day 1 average pain score**	**+ 0.26**	**0.05 to 0.46**	**0.01**
**Postop day 2 average pain score**	**+ 0.3**	**0.07 to 0.53**	**0.009**
Time to first ambulation, hours	+ 0.3 h	− 1.1 to 1.8	0.46
Urinary catheter duration, days	+ 2.4 days	− 2.3 to 7.3	0.43
**Time to ordering of solid food, days**	**− 0.6 days**	− **0.28 to** − **0.9**	**0.002**
	ODDS ratio*		
Return to operating room within 30 days	0.96	0.46 to 2	0.92
Readmission within 30 days	0.86	0.6 to 1.2	0.36

*Covariate of interest was post-implementation year (compared with pre-implementation year). Models accounted for age, gender, service line, American Society of Anesthesiologist (ASA) rating, opioid use at the time of admission (including methadone), surgical approach (minimally invasive vs open), case mix index, epidural use, and multimodal analgesic

## Discussion

Our study shows that by harnessing existing quality improvement infrastructure in surgery, relatively few additional resources can be invested and significant improvements can be had with regard to decreasing opioid exposure in surgical patients. Our multivariate analysis showed a decrease in intraoperative OME use, followed by no effect in the first 24 h, and then a consistent decrease until discharge. Discharge opioid prescribing was also reduced in the post-implementation year. The lack of decrease in OME use in the first 12 h likely represents rebound pain after suppression during the intraoperative phase. Although pain scores increased after the educational intervention, the difference may be a reflection of better patient education and expectation setting, as all patients had access to opioids in the postoperative orders if pain escalated. Importantly the goal of perioperative analgesia is to make the pain tolerable so that patients can comfortably complete their activities of daily living and then transition back to the outpatient setting. The goal is not to have a pain score of zero, and this was communicated by providers to patients at all phases of care.

Enhanced recovery programs are an important framework that many hospitals have in place where additional interventions around optimal analgesia and opioids exposure could be considered. Multimodal analgesia is a cornerstone of most enhanced recovery programs and likely one of the main elements that drive reduced length-of-stay and improved patient experience, but specific education about opioids and non-pharmacologic analgesia is not emphasized in most guidelines (Carmichael et al., 2017). While strong evidence supports multimodal analgesia, little is known about the true number of patients who are eligible for the full complement of medications, particularly non-steroidal anti-inflammatory medications and gabapentinoids, which in general are avoided in older patients. In addition, surgeon and physician buy-in varies significantly. Barriers have included patient and provider confusion about dosing and efficacy and reports about side effects that are worrisome in surgical populations. Examples include anastomotic leak and bleeding with non-steroidal anti-inflammatory agents (Modasi et al., 2018; Strom et al., 1996). Although we previously reported results of our program for colorectal (Sarin et al., 2016) and gynecology oncology (Chapman et al., 2016) and demonstrated uptake of multimodal analgesia, our current study shows that further uptake in multimodal analgesia prescribing and administration is challenging, and even with the multipronged approach outlined above, only 44% of the patients received multimodal analgesia postoperatively as defined by two or more non-narcotic medications administered. Despite this, we show that provider and patient education as well as non-pharmacologic adjuncts and an intraoperative analgesia protocol can help to reduce OME use intraoperatively and after postoperative day 1. This reduction persists till discharge and results in fewer opioid prescriptions sent out into the community as well as decreased risk of new persistent opioid use (Sun et al., 2016). We attribute this to decreased opioid prescribing, increased use of non-prescription analgesia strategies, and better patient education.

A major focus of our effort was on interns and residents, who are an integral component of care delivery at academic medical centers and often overlooked in hospital quality initiatives (Stone et al., 2016). They are important to engage because they are frequently first-line responders, particularly after hours, and armed with additional knowledge about analgesia (both pharmacologic and non-pharmacologic), they can effectively reduce opioid initiation or escalation. In addition, they represent the next generation of surgeons and anesthesiologists, and by actively engaging them, we are increasing the chance that this practice becomes standard of care.

Our study had several limitations. First, while the data was prospectively collected, there was a lack of randomization, which limits our ability to directly attribute opioid reduction to education alone. Second, the heterogeneity of surgeons, procedures, and diagnoses, while enhancing the generalizability of the study, prevents us from being able to analyze the impact of the intervention in more detail. Yet, the heterogeneity of the population is partly obviated by the relatively large and “real world” sample size. Third, in keeping with national trends, we noted a shift from laparoscopy to robotic surgery, but we would not expect this would affect the results. Finally, it was difficult to have an objective measure of education and therefore a process metric to track it; instead, we tried to target providers in all phases of surgical care and to have standard communications with them using information that was harmonized.

## Conclusion

Ultimately, sustainable success in reducing opioid analgesia for diverse surgical procedures will require a holistic approach (Chou et al., 2016). Our study demonstrates that with a targeted educational strategy that is concrete and sensitive to the role of different providers (surgeons, anesthesiologists, nurses), it is possible to further decrease postoperative OME use, even in mature surgical pathway programs. Addressing the opioid crisis will require a collaborative approach that combines education, regulation, and electronic health record tools aimed at maintaining pain control while stemming the flow of opioids into the community. This study demonstrates that concentrating on frontline provider education with a focus on setting patient expectations and improving knowledge regarding pain and opioids can be effective and that existing pathway or enhanced recovery programs are a promising area to focus on in surgery. It is likely that this strategy—engaging frontline providers and harnessing existing surgical pathway infrastructure—will also be helpful for the next, yet to be identified, crisis in surgical care.

## Supplementary information

**Additional file 1.** Established ERAS protocols

**Additional file 2.** Nursing Analgesic Resources Booklet

**Additional file 3.** Opioid conversion tables.

## Data Availability

The datasets used during the current study are available from the corresponding author on reasonable request.
