# Supplementary material for: Using provider-focused education toolkits can aid enhanced recovery programs to further reduce patient exposure to opioids
Source: Perioper Med (Lond). 2020 Jul 9;9:21. doi: 10.1186/s13741-020-00153-5 (PMC7346381; doi:10.1186/s13741-020-00153-5)
Supplement: Supplementary file 1 — Additional file 1. Established ERAS protocols [file 13741_2020_153_MOESM1_ESM.pdf]

			ANESTHESIA		SURGERY	NURSING	PATIENT
DAYS B4	PREPARE		Phone Consult or Appointment		Enter surgery & pre-op orders		Enroll in MyChart, Visit ERAS website for information,
			Deliver instructions via MyChart or mail.		Patient Education, EMMI videos		Prehabiliation: Follow Exercise program,
					Stoma marking and teaching		Clears liquids 7am and bowel prep noon on day before surgery
DOS. PRE-OP	MEDICATIONS		Pre-Op Warming. PIV. Crystalloid @ 30 ml/hr		Most patients get Oral and Mechanical Bowel preparation	Please complete Pre-Op RN checklist 45 minutes prior to OR start time, then Green Light.	Only clears day prior to surgery, NPO for fours hours before surgery except for a Boost Breeze completed 2 hours before coming to hospital.
		ANALGESICS	Gabapentin	600mg once	On clears day prior to surgery, Nothing by mouth for fours hours before surgery except for a Boost Breeze completed 2 hours before coming to hospital.		
			Acetaminophen	1000mg once		Apply Warming Blanket to patient. Teach ICS.	Risks of surgery and anesthesia will be discussed. You will sign a consent for the procedure, and discuss the possibility of receiving blood products.
			Diclofenac (if eGFR>60)	100mg once		IV Placed. Crystalloid started at 30ml/hr.	
		PONV	Scopolamine	1.5mg TD once			
			Age < 60 years		Consent checked, Site Marking, and 24-hr H&P completed 40 minutes before OR start time + link pathway. Discuss Epidural need with anesthesia team.	Gabapentin 600, APAP 1000, Diclofenac given once with water (<100ml). Antiemetics may also be ordered.	If there is any chance you might be pregnant, please discuss with surgery and anesthesia
	REGIONAL		30 minutes before start time, complete anesthesia assessment, go to Block Room, and place Thoracic Epidural placed at T8-10				
INTRA-OP			Maintain patient temperature >36				
			Goal FSBG < 180				
			Orogatric tube to low intermittent suction		If on steroids, ask for Hydrocortisone 100mg IV x 1		
	Medications	VTE	Heparin 5000 U SC X 1 after epidural placement				
		IVFs	Fluids: NTE 2 L for straightforward colectomies unless EBL>300ml,				
		ABX	Antibiotic: 1 g ceftriaxone + 500 mg metronidazole IV				
		PONV	Dexamethasone	4mg IV x 1 after induction			
			Metoclopramide 10mg IV X 1. Unless contraindicated.				
			Ondansetron	4mg IV x 1			
			Propofol gtt (with >3 RFs)				
	Pain Management	ALL	Minimize opioid medications				
			If Opioid-Tolerant, continue their opioid regimen intra-op. Start ketamine load and infusion. 0.2 mg/kg x 1. Then 2 mcg/kg/min.		ERAS TIMEOUT: Review opioid sparing stategy, PONV, SCIP measures + IVF management		
			IV Toradol 30 mg (to be confirmed at timeout)				
		Laparoscopic	If epidural deferred, lidocaine gtt @ 2 mg/kg/hr		If epidural deferred, surgeon infiltration 0.25% bupivicaine		
			Magnesium bolus 30 mg/kg (over 30 minutes) then 6 mg/kg/hr				
		Ostomy	Lidocaine and magnesium gtt		Surgeon infiltration 0.25% bupivicaine		
		Open	Thoracic Epidural 0.0625% Ropi + Fentanyl 2 mcg/ml @ 8 ml/hr (if not amenable to epidural, then consider TAP vs lido/mg gtt.		ERAS Debrief: Post-op pain regimen, diet orders, heparin dosing		

PACU	MEDICATION	Minimize opioid medications		Continue orders on Colorectal Surgery Pathway Orderset	Minimize opioid medications	
		Order Antiemetics		Choose Famotidine (if GERD or steroids)	Thoracic Epidural 0.0625% Ropi + Fentanyl 2 mcg/ml @ 8 ml/hr	
	REGIONA	Thoracic Epidural 0.0625% Ropi + Fentanyl 2 mcg/ml @ 8 ml/hr		Choose Toradol if appopriate		
FLOOR/ICU POD 0	MEDICATIONS	Gabapentin	600mg PO QHS	Immediate Post op Labs on select patients only	Vital Signs q 4H, I&O shift, weight daily, surgical incision care abdomen,	Out of bed 6 hours after surgery
		Acetaminophen	1000mg IV q6H	Limited clear diet 500ml per shift	Out of bed 6 hours after surgery with assistance of Nursing	Incentive Spirometry x10 q 1H
		Toradol (if eGFR>60)	15mg IV q6H	Address stoma care and ileostomy teaching	Encourage Incentive Spirometry x10 q 1H	Limited Clears (<500 ml per shift)
		If Opioid-Tolerant, continue ketamine infusion 2 mcg/kg/min and maintain daily opioid req.		Address delirium precautions	Foley Catheter to gravity.	Gum Chewing encouraged
		IV Dilaudid and Oxycodone PRN		Goal FSBG < 180	DVT Proph: Heparin 5kU SQ TID	
	REGIONAL	Thoracic Epidural 0.0625% Ropi + Fentanyl 2 mcg/ml @ 8 ml/hr				
FLOOR/ICU POD 1-2	MEDICATIONS	Gabapentin	600mg PO QHS	Evaluate IV Fluids and avoid hypervolumia	Vital Signs q 4H, I&O shift, weight daily, surgical incision care abdomen,	Walking 5 times a day. At least first time with nurse.
		Acetaminophen	1000mg IV/PO q6H	Labs: CBC, Cr, BUN	Ambulation: OOB to chair (3hrs) BID<br>Ambulation 5 x per day	Incentive Spirometry x10 q 1H
		Toradol OR Diclofenac (eGFR)	15mg IV q6H<br>50mg PO TID	Unlimited clears on POD 1. On POD2 Regular diet /Low residue for new ileostomies ileoanals	Encourage Incentive Spirometry x10 q 1H	Unlimited Clears or Regular / Low residue diet
		If Opioid-Tolerant, continue ketamine infusion 2 mcg/kg/min and maintain daily opioid req.		Address Foley removal either today o POD 4 (if pelvic dissection)	Remove Foley Catheter in AM	Gum Chewing OK
		IV Dilaudid and Oxycodone PRN		Address steroid taper if appropriate	DVT Proph: Heparin 5000U SQ TID	
	REGIONAL	Thoracic Epidural Continue POD1. Stop infusion at 6AM on POD2. Catheter to be removed		Hold 6AM Heparin dose for epidural removal POD2		
FLOOR/ICU Discharge	MEDICATIONS	Tylenol		Meds to Beds	Discharge Teaching	Ensure questions answered
		Ibuprofen		Clear discharge instructions with use of adjunct nonopioid pain meds	Meds to Beds	Check Follow up appointment date and time
		Opioid		Plan for staples, drains, follow up labs appointment in place		Confirm plan in place for drains, staples pain meds, other meds, follow up
						Have support at home in place for discharge
	NAL					
						Edited: 3/2017

UCSF Gyn Onc Enhanced Recovery Pathway

			ANESTHESIA	GYN ONC	NURSING	PATIENT
DAYS B4		PREPARE	Phone Consult or Appointment	Informed consent.	TUG assessment	Enroll in MyChart
			Provide pre-op instructions via MyChart or mail	Patient education, expectations management, Exercises for Recovery, Consider Surgical Wellness		Learn about post-op goals and expectations, acquire Boost breeze and antibiotics for bowel prep as indicated. Consider Impact or other immunomoduation for vulvectomy or open surgical cases
				Enter pre-op orders: medications, T&S/T&C, albumin (open cases)		Complete Exercises for Enhanced Recovery
Day of Surgery / Pre-op			Pre-Op warming, PIV, LR at 30 ml/hr.	Consent checked and 24Hr H&P completed 45 minutes before OR start time	Complete Pre-Op RN checklist 45 min prior to OR start time	No food after midnight. Boost breeze or equivalent carbohydrate drink (e.g. Gatorade) and water taken up until 2 hours before arrival to hospital on day of surgery.
			Complete anesthesia assessment		Pre-Op warming, IV placed, labs drawn (if applicable), LR at 30mL/hr, ISS teaching	
		MEDICATIONS		Gabapentin 600mg once<br>Acetaminophen 1000 mg PO once<br>Diclofenac (eGFR>60) 100mg PO once<br>Scopolamine (age <60) 1.5mg TD once (if > 3RFs)	Gabapentin, diclofenac & APAP given once with sip of water	Risks of surgery and anesthesia will be discussed. You will sign a consent for the procedure, and discuss the possibility of receiving blood products. You will also have the option of consenting to tissue banking.
	REGIONAL		30 min before start time, place T9-10 epidural (open cases)			
INTRA-OP			Draw 4 purple top tubes if consented for tissue bank	ERAS TIMEOUT: Review opioid sparing strategy, PONV, SCIP measures, IVF management, need for clean closing tray (if open clean contaminated or contaminated case), tissue banking		
			Orogastric tube to low intermittent suction (laparoscopic cases)			
			Maintain patient temperature >36.0 C			
	MEDICATIONS	ABX	Antibiotic: Cefazolin 2-3g IV q4			
		IVF	Fluids: NTE 2L unless EBL >300mL. Use esophageal doppler or SPV (+ a-line) to guide resuscitation			
		PONV	Dexamethasone 4mg IV x 1 after induction/before incision<br>Ondansetron 4mg IV x 1<br>Propofol gtt (if > 3RFs)			
	PAIN MANAGEMENT	All	Minimize opioid medications	ERAS Debrief; Post-op pain regimen, diet orders, heparin dosing		
		Lap	*Lidocaine 2mg/kg/hr IV + Magnesium 30 mg/kg bolus over 30 minutes followed by 6 mg/kg/hr*			
		Open	*Thoracic epidural Ropiv 0.0625% + Fentanyl 2 mcg/mL @ 8 mL/hr. Lidocaine + Magnesium drips for patients who are not candidates for epidural.*			
	END OF CASE		Alveolar recruitment maneuver: sustained inflation by CPAP with pressures from 30 to 40 cmH2O for 30 seconds x 3 IMMEDIATELY prior to extubation (laparoscopic cases)	Request Alveolar recruitment maneuver for laparoscopic cases	Provide clean closing tray and new gloves if open case	

UCSF Benign Gynecology ENHANCED RECOVERY PATHWAY

			ANESTHESIA		Gyn MD	NURSING	PATIENT
DAYS B4	PREPARE		Verify pre-op meds ordered by Gyn (see below)		Use orderset #2122: med orders & instructions re: TAP block in case booking comments		Learn post-op goals and plans for discharge.
			Phone or in person consult: provide pre-op instructions via MyChart (".PREPAREERAS")		Patient Education, hand out brochure		
DOS. PRE-OP			Order Pre-Op Warming. PIV. Crystalloid @ 30 ml/hr		No mechanical bowel prep prior to arrival	Please complete Pre-Op RN checklist 45 minutes prior to OR start time, then Green Light.	Solid food allowed until day before surgery. Clear liquids taken up until 2 hours before arrival
	MEDICATIONS	ANALGESICS	Gabapentin	600mg once	On clears day prior to surgery, Nothing by mouth for fours hours before surgery except for a Boost Breeze completed 2 hours before coming to hospital.		
			Acetaminophen	1000mg once		Place Pre-Op warming with bear hugger. IV placed. Crystalloid at 30mL/hr.	Risks of surgery and anesthesia will be discussed.
			Diclofenac (if eGFR>60)	100mg once			
		PONV	Scopolamine	1.5mg TD once (if > 3RFs)		Urine Preganancy Test	
					Consent checked, Site Marking, and 24-hr H&P completed 40 minutes before OR start time. Discuss Epidural need with anesthesia team.	ICS teaching	
	REGIONAL					Gabapentin 600, APAP 1000, Diclofenac given once with water (<100ml).	
INTRA-OP			Maintain patient temperature >36 C		TIMEOUT: Review opioid sparing stategy, PONV, SCIP measures + IVF management		
			Alveolar recruitment maneuver: sustained inflation by CPAP with pressures from 30 to 40 cmH2O for 30 seconds x 3 IMMEDIATELY prior to extubation. (laparoscopic cases)		Request Alveolar recruitment maneuver		
			Orogastric tube inserted for laparoscopic surgery, placed to suction once and then clamped.		If on steroids, ask for Hydrocortisone 100mg IV x 1		
	Medications	VTE	All cases: SCDs; Open Cases with risk factors: Heparin 5000 U				
		TXA	TXA 10 mg/kg bolus => 1 mg/kg/hr For EBL>400		Request TXA for expected EBL>400		
		IVFs	Fluids: NTE 2 L for straightforward colectomies unless EBL>300ml,				
		ABX	Antibiotic if indicated (mainly for hysterectomy): Cefazolin	1-2g IV q4			
		PONV	Dexamethasone	4 mg IV x 1 after induction/before incision			
			Ondansetron	4mg IV x 1			
			*Metoclopramide	10mg IV x 1 (if > 3RFs)			
	PAIN Medication	ALL	Minimize opioid medications				
		Laparoscopic	*IV lidocaine 2mg/kg/hr *IV magnesium 30 mg/kg bolus over 30 minutes followed by 6 mg/kg/hr (For lap or if patient or provider declines TAP)				
		Open	*Bilateral TAP block. 20ml of Ropi 0.2% each side		DEBRIEF: Review opioid sparing strategy, diet advancement, fluids		
ACU	CATION		Minimize opioid medications. Order opioid of choice: Hydromorphone or Morphine		Post-op Orderset #2345	Minimize opioid medications. Hydromorphone or Morphine IV PRN. Titrate to RR 12.	

PA	MEDI	Order Antiemetics				
		GYNECOLOGY SERVICE			NURSING	PATIENT
FLOOR/ICU POD 0	MEDICATIONS	Gabapentin	600mg PO QHS	Determine potential discharge POD1 - plan Discharge Orders	Vital signs q4H, I&O shift	Out of bed 6 hours after surgery (if patient awake)
		Acetaminophen	1000mg PO or IV q6H		Fluids: Maintenance IVF for 6 hrs post-op, then SLIV Diet: Regular.	Diet: Regular
		*Toradol (if eGFR>60)	15mg IV q6H		Activity: OOB to chair @ 6 hrs post-op	Ambulation: ASAP
		*Diclofenac (if eGFR>60)	50mg PO BID		GI ppx: Senna & Colace	Keep SCD boots on
		IV Dilaudid and Oxycodone PRN			Foley catheter out at 6 hours post-op	
FLOOR/ICU POD1 -	MEDICATIONS	Continue pain regimen while in-patient		No routine labs	Discharge Teaching	Ambulation ASAP
		Discharge home with Colace 250mg BID prn constipation		Discharge order in before 10am to facilitate Discharge by Noon	Discharge by Noon	Plan transportation to be ready before noon
		Home pain regimen: Acetaminophen, ibuprofen and oxycodone				

UCSF CYSTECTOMY ENHANCED RECOVERY PATHWAY

		ANESTHESIA		SURGERY	NURSING	PATIENT
DAYS B4	PREPARE	Phone Consult: deliver instructions via MyChart or mail.<br>Instructions: ".prepareeras"		Informed Consent. Enter pre-op orders (**#1356**)		Enroll in MyChart
				Hand out brochure<br>.erascystectomy		Option for prehab etc.
DOS. PRE-OP	MEDICATIONS	Pre-Op Warming. PIV. Crystalloid @ 30 ml/hr		No bowel prep needed unless colonic diversion planned (rare). Fleets Enema can be perfomed the AM of surgery if necessary based on surgeon preference	Please complete Pre-Op RN checklist 45 minutes prior to OR start time, then Green Light.	Nothing by mouth for eight hours before surgery except for clears/ Boost Breeze completed 2 hours before coming to hospital (Arrival Time)
		Entereg (Alvimopan 12 mg))	1 tab po once prior to surgery (30min to 5 hours prior)			
	ANALGESICS	Gabapentin	600mg once	Nothing by mouth for eight hours before surgery except for clears/ Boost Breeze completed 2 hours before coming to hospital (arrival time)		
		Acetaminophen	1000mg once		Apply Warming Blanket to patient. Teach IS.	Risks of surgery and anesthesia will be discussed. You will sign a consent for the procedure, and discuss the possibility of receiving blood products
		Diclofenac (if eGFR>60)	100mg once		IV Placed. Crystalloid started at 30ml/hr.	
	PONV	Scopolamine<br>Age < 60 years	1.5mg TD once		Give appropriate preoperative medications (see anesthesia)	
	REGIONAL	Consider thoracic Epidural placed at T8 10 per surgeon preference		Consent checked, Site Marking, and 24-hr H&P completed 40 minutes before OR start time.		If there is any chance you might be pregnant, please discuss with surgery and anesthesia
INTRA-OP	VTE	Heparin 5000 U SQ X 1 (after epidural placement- if epidural used)		Epidural placement per surgeon preference vs local infiltration vs PCA post operatively		
	OG	Orogastric tube to low intermittent suction.				
	IVFs	Fluids: NTE 2L unless EBL>300ml, Esophageal doppler monitoring. Minimize fluids especially during ureteral clamping				
	Temp	Patient temperature must not drop below 36.0 C.				
MEDS	ABX	Antibiotic: Ceftriaxone + Flagyl	Aztreonam + flagyl or Ertapenem (2nd choice)			
	Pain	Minimize opioids administration				
		If Opioid-Tolerant, continue their opioid regimen intra-op. Start ketamine load and infusion. 0.2 mg/kg x 1. Then 2 mcg/kg/min.				
	PONV	Dexamethasone	4mg IV x 1 after induction			
		Metoclopramide 10mg IV X 1. Unless contraindicated.				
		Ondansetron	4mg IV x 1			
	REGIONAL	Thoracic Epidural 0.0625% Ropi + Fentanyl 2 mcg/ml @ 8 ml/hr (if not amenable to epidural, then consider TAP vs lido/mg gtt.		Local anesthetic with bupivicaine if no epidural. Prior to skin clsoureby supra and sub-facial injection.		

Edited March 2017

Phase	Category	ANESTHESIA		SURGERY	NURSING	PATIENT
PACU	MEDICATION	Minimize opioids ordered		Post-op Orderset **#1358**	Minimize opioids administered	
PACU	MEDICATION	Order Antiemetics		Consult APS if OME>100	Thoracic Epidural 0.0625% Ropi + Fentanyl 2 mcg/ml @ 8 ml/hr	
PACU	REGIONAL	Thoracic Epidural 0.0625% Ropi + Fentanyl 2 mcg/ml @ 8 ml/hr				
FLOOR/ICU POD 0	MEDICATIONS	Gabapentin	600mg PO QHS	Alvimopan 1 tab PO BID, not to exceed 14 doses or 7 days pm dose should be given on POD#0	Vital Signs q 4H, I&O shift, weight daily, surgical incision care	
FLOOR/ICU POD 0	MEDICATIONS	Acetaminophen	1000mg IV q6H		Ambulation: OOB ad lib, attempt x 1 evening	Out of bed ad lib, x 1 evening of surgery
FLOOR/ICU POD 0	MEDICATIONS	Toradol (if eGFR>60)	15mg IV q6H	Address delirium precautions	Incentive Spirometry x15 q 1H	Incentive Spirometry x15 q 1H
FLOOR/ICU POD 0	MEDICATIONS	If Opioid-Tolerant, continue ketamine infusion 2 mcg/kg/min and maintain daily opioid req.		Gaols FSBG<180	Foley Catheter to gravity (if neobladder) vs stoma bag to gravity	
FLOOR/ICU POD 0	MEDICATIONS	If needed, PCA HM 0.2/10/0			DVT Proph: Heparin 5kU SQ TID	
FLOOR/ICU POD 0	REGIONAL	Thoracic Epidural 0.0625% Ropi + Fentanyl 2 mcg/ml @ 8 ml/hr			Gum chewing ok. NPO vs sips per surgeon	Gum chewing ok
FLOOR/ICU POD 1	MEDICATIONS	Gabapentin	600mg PO QHS	Alvimopan 1 tab PO BID, not to exceed 14 doses or 7 days	Vital Signs q 4H, I&O shift, weight daily, surgical incision care	
FLOOR/ICU POD 1	MEDICATIONS	Acetaminophen	1000mg IV/PO q6H	Labs: CBC, Cr, BUN	Ambulation: OOB to chair (3hrs) BID Ambulation 5 x per day	Walking 5 times a day. At least first time with nurse.
FLOOR/ICU POD 1	MEDICATIONS	Toradol OR Diclofenac	15mg IV q6H/ 50mg PO TID		Incentive Spirometry x15 q 1H	Incentive Spirometry x15 q 1H
FLOOR/ICU POD 1	MEDICATIONS	If Opioid-Tolerant, continue ketamine infusion 2 mcg/kg/min and maintain daily opioid req.		Wound RN consult for stoma care vs neobladder irrigations	Wound RN consult for stoma care vs neobladder irrigations	
FLOOR/ICU POD 1	MEDICATIONS	If needed, PCA HM 0.2/10/0		Consider reglan if persistent nausea	DVT Proph: Heparin 5kU SQ TID	
FLOOR/ICU POD 1	REGIONAL	Thoracic Epidural 0.0625% Ropi + Fentanyl 2 mcg/ml @ 8 ml/hr			sips vs clears per surgeon if no sx of ileus, Gum chewing ok.	Gum chewing ok
FLOOR/ICU POD ##	MEDICATIONS	Gabapentin	600mg PO QHS	Alvimopan 1 tab PO BID, not to exceed 14 doses or 7 days	Vital Signs q 4H, I&O shift, weight daily, surgical incision care	
FLOOR/ICU POD ##	MEDICATIONS	Acetaminophen	1000mg IV/PO q6H	Nutrition Consultation	Ambulation: OOB to chair (3hrs) BID Ambulation 5 x per day	Walking 5 times a day. At least first time with nurse.
FLOOR/ICU POD ##	MEDICATIONS	Toradol OR Diclofenac	15mg IV q6H/ 50mg PO TID		Incentive Spirometry x15 q 1H	Incentive Spirometry x15 q 1H
FLOOR/ICU POD ##	MEDICATIONS	If Opioid-Tolerant, continue ketamine infusion 2 mcg/kg/min				
FLOOR/ICU POD ##	MEDICATIONS	If Opioid-Tolerant, continue their daily opioid requirement.			DVT Proph: Heparin 5kU SQ TID	
FLOOR/ICU POD ##		PCA HM 0.2/10/0 vs Advance to po opioids when tolerating PO		Advance to po opioids when tolerating PO		
FLOOR/ICU POD ##	REGIONAL	Epidural should not be weaned but shut off once appropriate oral meds initiated			Clear liquid diet or diet advance. SLIV and support with small NS bolus as needed	Clear liquid diet or diet advance.

Edited March 2017
