# Supplementary material for: Using provider-focused education toolkits can aid enhanced recovery programs to further reduce patient exposure to opioids
Source: Perioper Med (Lond). 2020 Jul 9;9:21. doi: 10.1186/s13741-020-00153-5 (PMC7346381; doi:10.1186/s13741-020-00153-5)

# References


- Fang, M., Kayser, S., & Naidu, R. (2015). Guidelines for the Use of Antithromboitic Agents in the Setting of Neuraxial Procedures. Retrieved from

http://medctrpharm.ucsf.edu/system/files/documents/UCSF%20Antithrombotics%20%20Neuraxial%20Intervention%20July%202015.pdf

- Lexicomp. Wolters Kluwer Clinical Drug Information, Inc. Retrieved from

http://www.crlonline.com/lco/action/home

- Pasero C & McCaffery M. (2011). *Pain assessment and pharmacologic management*. St. Louis: Mosby/Elsevier.
- UCSF Nursing Procedure: Epidural Analgesia (General)

http://manuals.ucsfmedicalcenter.org/NursingDept/AdultProcedures/PDFsafter12-29-2003/EpiduralAnalgesia_General_.pdf

- UCSF Nursing Procedure: Ketamine Low Dose Intravenous Administration on Acute Care Units and in the Emergency Department (General)

http://manuals.ucsfmedicalcenter.org/NursingDept/AdultProcedures/PDFsafter12-29-2003/KetamineLowDoseIVInfusionOnDesignatedAcuteCareUnits_General.pdf

- UCSF Nursing Procedure: Pain Assessment (General)

http://manuals.ucsfmedicalcenter.org/NursingDept/AdultProcedures/PDFsafter12-29-2003/PainAssessment_General_.pdf

- UCSF Nursing Procedure: Patient Controlled Analgesia (Intravenous) (General) http://manuals.ucsfmedicalcenter.org/NursingDept/AdultProcedures/PDFsafter12-29-2003/PatientControlledAnalgesia_General_.pdf
- UCSF Nursing Procedure: Peripheral Nerve Catheters, Local Anesthetic Infusion Via (General) http://manuals.ucsfmedicalcenter.org/NursingDept/AdultProcedures/PDFsafter12-29-2003 /PeripheralNerveCathetersLocalAnestheticInfusionviaGeneral.pdf
- UCSF Nursing Procedure: Range Orders for Pain Management (General)

http://manuals.ucsfmedicalcenter.org/NursingDept/AdultProcedures/PDFsafter12-29-2003/RangeOrdersforPainManagement_General_.pdf




# Pain Guide

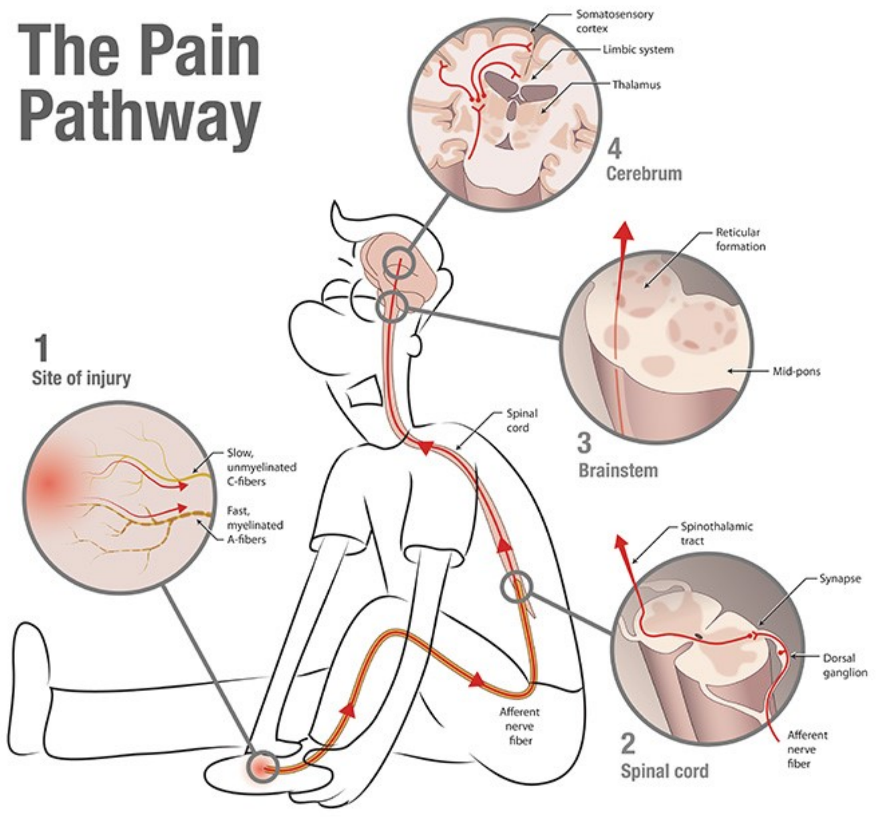


http://www.feelingprettyremarkable.com

## UCSF MISSION BAY

## Adult Acute Care 5 & 6


J. Kulwin 5/2018

Reviewed by S. Brynelson, Pain CNS

# Methadone

- Multiple mechanisms of action make it unique alternative
- Long but variable half-life, watch for accumulation & toxicity
- Duration of analgesic effect = 22-48 hours once steady state achieved (at least 3-5 days)
- May be used in both renal & hepatic impaired patients
- Large number of medication interactions to consider, also avoid grapefruit interaction
- Potential for cardiac arrhythmia—may need routine ECGs
- Instruct patient to talk to their doctor about methadone reduction before stopping to avoid withdrawal reactions

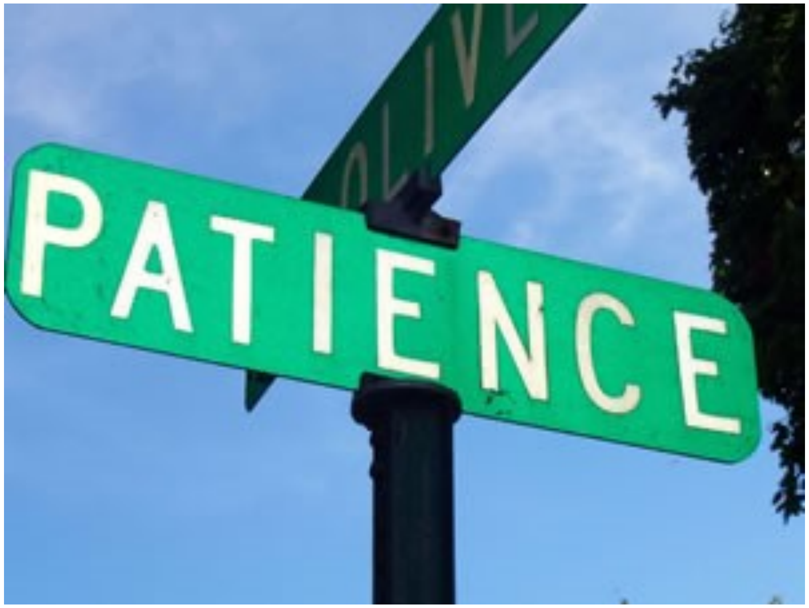


http://ahsdavishonors.wikispaces.com/

# Common Pain Medications

## Tramadol (Ultram)

- Atypical opioid treats moderate to moderately severe pain
- Dual mechanism of action: weak bind to mu opioid receptor and inhibits serotonin/norepinephrine re-uptake
- Most common side effects: nausea/vomiting, dizziness, drowsy, dry mouth. Less risk of respiratory depression.
- Consider drug interactions:  SSRIs may inhibit metabolism, so caution for serotonin syndrome and/or history seizures

## Gabapentin (Neurontin)

- Used for first-line neuropathic pain and acute perioperative pain to improve analgesia, allows lower doses than other analgesics, helps prevent persistent neuropathic pain syndromes
- Most dose-limiting side effects: sedation & dizziness
- Not metabolized in liver so minimal drug interactions, but caution in renal impairment



# Table of Contents

Introduction ........................................................1
Assessment .......................................................2-4
Individualized Pain Treatment ............................5
Range Orders .....................................................6
Multimodal Analgesia .......................................7
High Risk Medication Management ...................8
Patient Controlled Analgesia (PCA) ...................9
Epidural Analgesia ......................................10-11
Peripheral Nerve Infusion ................................12
Naloxone (Narcan) ...........................................13
Ketamine ....................................................14-15
Guidelines for Antithrombotic Agents ......16-17
Common Pain Medications .......................18-19
References .......................................................20

# Guidelines for Antithrombotic Agents in Neuraxial Procedures

| *FOR VENOUS THROMBO-EMBOLISM PROPHYLAXIS* | **Minimum time after catheter placement to drug start** | **Minimum time between last dose of drug & catheter removal** | **Minimum time between catheter removal & when next dose can be given** |
|---|---|---|---|
| Enoxaparin 40mg SQ q day | CAUTION: May be given, wait 6 hours after placement before next dose | 12 hours | 4 hours |
| Enoxaparin 30mg or 40 mg SQ bid | CONTRAINDICATED while catheter in place | CONTRAINDICATED while catheter in place | 4 hours |
| Heparin 5000 units SQ bid | May be given, no time restrictions | May be given, no time restrictions | May be given, no time restrictions |
| Heparin 5000 units SQ tid | 2 hours | 4 hours | 2 hours |

Find complete table & more information online:

Go to UCSF CareLinks and click Clinical Guidelines, Pain Management, Antithrombotics Neuraxial Intervention Guidelines July 2015

Or click http://medctrpharm.ucsf.edu/system/files/documents/UCSF%20Antithrombotics%20%20Neuraxial%20Intervention%20July%202015.pdf

# Should I GIVE or HOLD

# ANTICOAGULATION

# if my patient has an EPIDURAL?

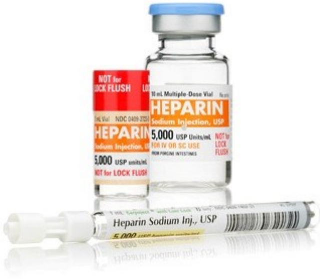


http://www.jacknaimsnotes.com/

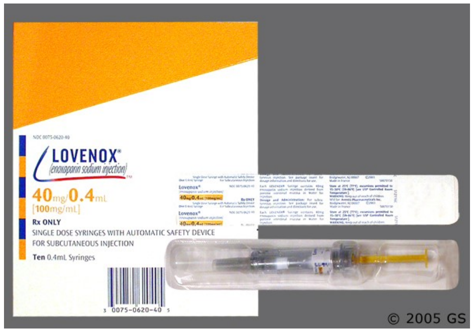


medconnections.com



## Introduction

Dear Nurses,

This study guide is a resource for the most relevant pain issues, information, and policies relevant to our unit.

Please use this as an education tool and resource in your daily practice.  This guide will be periodically updated.

For more questions, issues, or concerns please talk to your pain resource nurses:  Jessica Kulwin, Chonel Amores, and Cosima Singleton.  We are your peer resource for anything related to pain and are happy to support you!

# Assessment

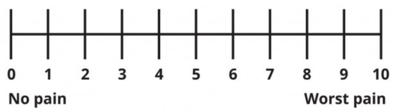


## Self Report

*Gold Standard*

Numerical, FACES, Verbal Descriptor

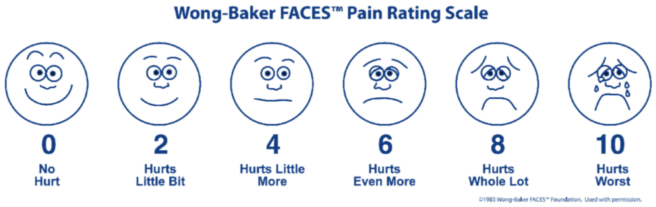


## Behavioral

CNPI (Checklist Non-Verbal Pain Indicators) for cognitively impaired

FLACC (Face, Legs, Activity, Cry, Consolability) for non-verbal

*Use only when patient can't self report*

Behavior pain score does not equal pain intensity

## Admission Assessment

Pain history in Admission Navigator:

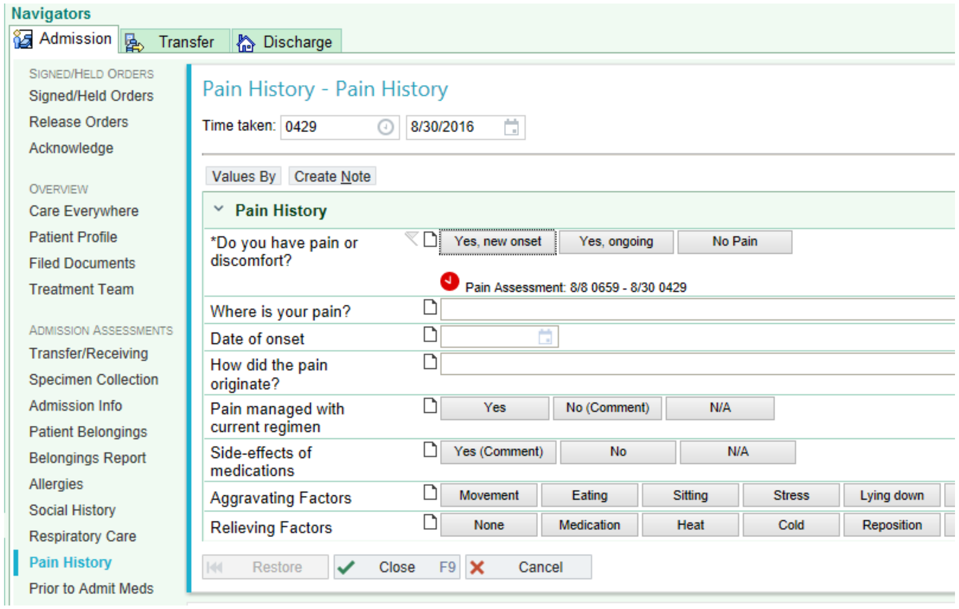




## Requirements

**Dedicated IV line with maintenance fluid,** if not available then need order for non dedicated line with compatible solutions

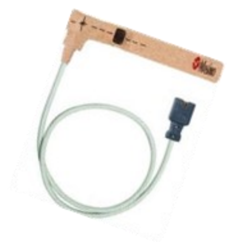

CPO at all times

Bag & tubing changed q 24 hours due to 1/2 life

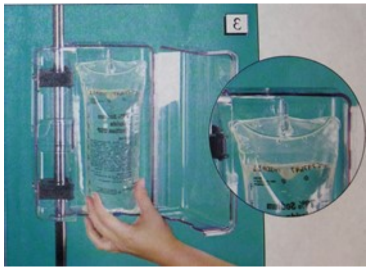

Portless IV tubing

Waste in pyxis under ALL meds, select IVAS NARC

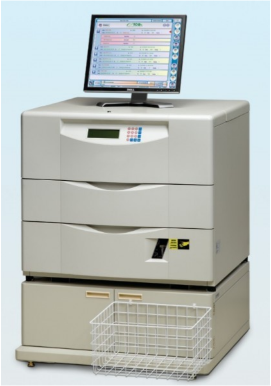

## Contraindications

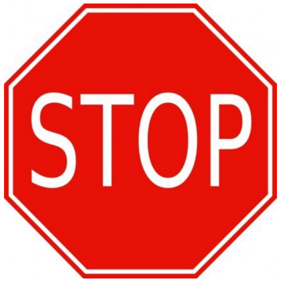


History of psychosis, delirium, recent seizures, labile hypertension, poorly controlled cardiac arrhythmia or intracranial hypertension

# Ketamine

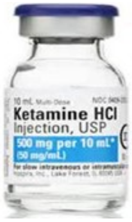


rebelem.com

LOW DOSE INTRAVENOUS for Acute Care

- Used primarily as adjunct treatment to manage severe intractable pain and produce overall opioid dose-sparing effect, so patient will need lower dose of concurrent opioids
- Only Pain Service or Palliative care may write orders BUT emergency stop orders can be from any provider

## Dose

- Low dose = $\leq$ 5 mcg/kg/min
- No range or titrating orders
- Can last 2.5 hours once discontinued, no weaning process necessary
- Up to 10 mcg/kg/min for patients on adult palliative care service

## Side Effects

- Lightheadedness, tired, dizzy
- Hypotension or hypertension, tachycardia
- Vivid dreams, hallucinations, irrational
- Hypersalivation, nausea

Small reduction in dose often resolves side effects while retaining analgesic benefit

No reversal agent available: stop infusion, notify provider, continue monitoring, and treat as appropriate



# Detailed Pain Assessment

Conduct every shift and at assumption of care for each new pain finding or event

*Acceptable goal* is based on functional status/history & agreed to in discussion with patient. Zero is not realistic after surgery.

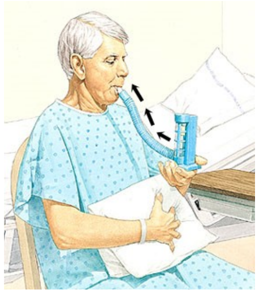

http://www.fairview.org/healthlibrary/Article/86596

***"What does your pain level need to be at for you to take deep breaths comfortably?"***

Includes all rows in pain assessment flowsheet and sedation level:

| **Pain Assessment** | | |
|---|---|---|
| Pain Assessment | | |
| Pain Level | | |
| Acceptable Level of Pain | | |
| Pain Type | | |
| Pain Location | | |
| Pain Orientation | | |
| Pain Character | | |
| Effect of Pain on Daily Activities | | |
| Multiple Pain Sites | | |
| **Pain Interventions** | | |
| Pain Interventions | | |
| [illegible] | | |

## Focused Pain Assessment

- After completing detailed pain assessment conduct before/ after any pain treatment & with vital signs
- Evaluate presence of pain, pain score, sedation level

## Reassessment Timing

*Timed around medication peak time*

- PO/PR/G tube: within **90** mins
- IV/IM/SQ/Epi within **30** mins
- Non-pharmacologic: within **90** mins

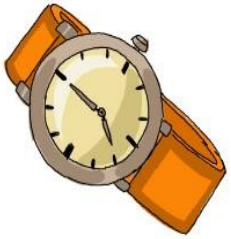

www.drawingnow.com

Q: Do I wake Sleeping Patient??

A: IT DEPENDS

- Involve patient in plan for managing pain while asleep
- If patient on stable pain regimen with good respiratory status then acceptable to allow sleep, BUT must conduct comprehensive respiratory assessment (depth, regularity, rate, noisiness) and awaken patient if any concern for over-sedation

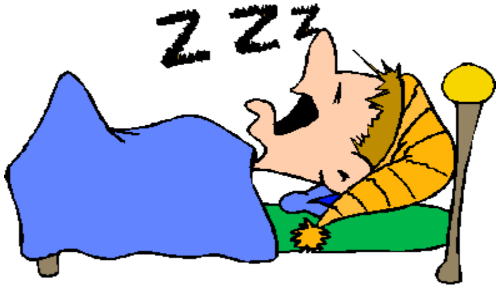


serinazzz.exteen.com

- Best practice is to wake patients for ATC pain meds



# Naloxone (Narcan)

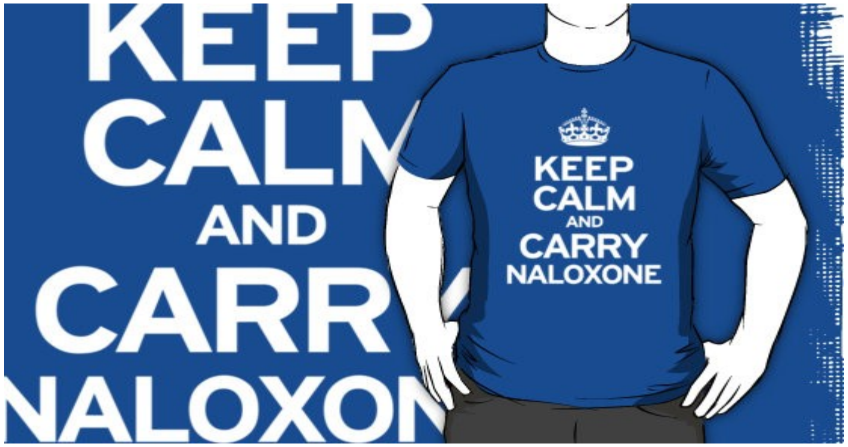


http://www.injectingadvice.com/v4/index.php/tshirts

- Opioid antagonist reverses respiratory depression & can be used to treat pruritus
- Used in 911 situation of severe respiratory depression (RR less than 8/min) or unresponsiveness (sedation level POSS =4)
- Included in PCA & Epidural order sets as PRN orders
- IV administration: rapid onset=2 minutes, peak=10 minutes, duration 1-4 hours, dilute in 10 ml NS, push slowly at rate of 0.5 ml every 2 minutes while observing patient's response

→ TITRATE to EFFECT

- Titrate carefully as too much or too fast can lead to severe pain & increase in sympathetic activity
- Multiple doses may be necessary because naloxone has shorter duration than most opioids
- Notify appropriate teams & **complete incident report** when given

# Peripheral Nerve Infusion

- Provides continuous infusion of local anesthetic (usually Ropivacaine) adjacent to peripheral nerve to block pain transmission to central nervous system
- Most commonly the sciatic, saphenous, or femoral nerves
- Similar to epidural administration through CADD Solis pump as continuous or PIB delivery, monitoring requirements, assessment of insertion site and tubing/catheter connection

## WHAT IS DIFFERENT?

- Solution labeled for “PERIPHERAL NERVE INFUSION”
- Monitor for signs of local anesthetic toxicity
    - Early: perioral numbness, tingling, blurry vision
    - Late: sedation, altered mental status, seizure, dyspnea, low or high blood pressure/heart rate, arrhythmias

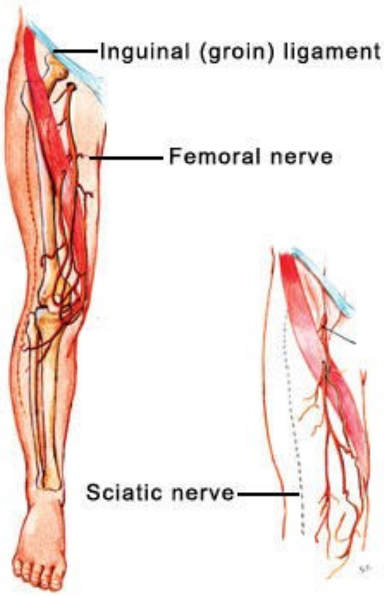


http://marilynbrooksonmedicine.com/

Compared to opioid analgesia

- superior anesthesia
- fewer side effects
- greater patient satisfaction



# Individualized Pain Treatment

*Many factors to consider when choosing which pain medicine, route, and dose in addition to “mild, moderate, or severe” pain intensity!*

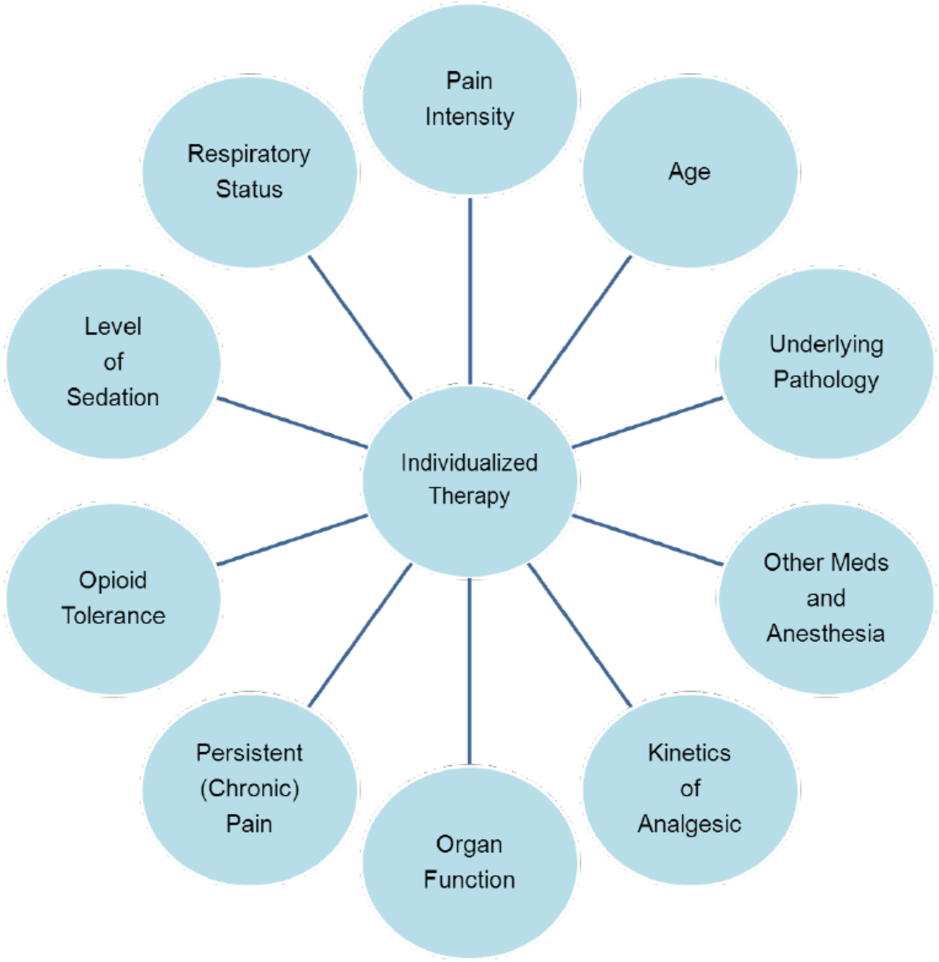


http://manuals.ucsfmedicalcenter.org/NursingDept/AdultProcedures/PDFsafter12-29-2003/PainAssessment_General_.pdf

# Range Orders

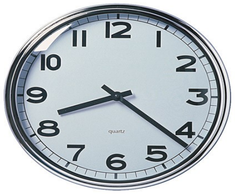


- Start with lowest dose unless prior knowledge that patient managed with higher dose
- If starting with higher dose of range, must document the reason
- Use “rolling clock” method to determine available dosage: ordered time interval is based on time of the last dose. Calculate how much has already been given over the ordered time interval since total dose cannot exceed the high end of the dose

  Example: Oxycodone 5-10mg every 4 hours PRN moderate pain

  0800 Patient’s pain score is 6/10 and receives 5 mg

  1000 Patient pain score is 5/10 and receives 5 mg

  1201 Patient’s pain score is 5/10 –in last 4 hours (from 801-1201) patient received 5 mg so now can only get another 5 mg at this time; giving the patient 10 mg now would mean the patient received 15 mg in last 4 hours and would exceed the dose range.
- Use *dose frequency* to determine how often additional doses can be given based on medication peak effect times
  - PO/PR/gastric tube analgesics: 60 mins
  - IV analgesics: 15-30 mins

  Example: Hydromorphone 0.2-0.6 mg IV every 2 hours PRN severe pain

  At 1400 0.2 mg IV was given. If additional doses are needed before reaching high end of the dose range, wait at least 15-30 minutes



Assess depth at insertion site

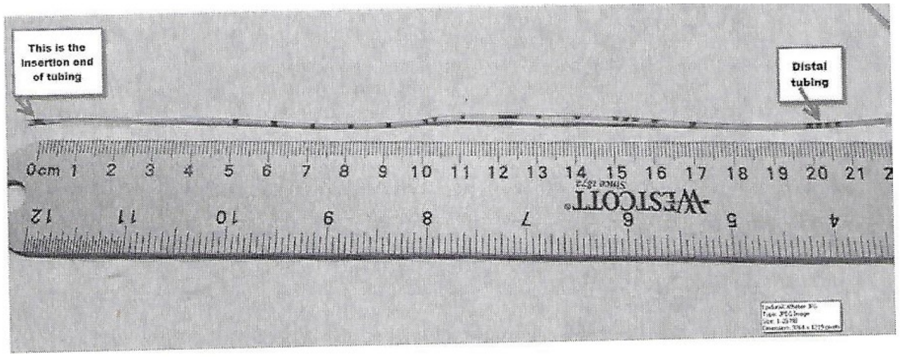


- The epidural catheter has hash marks 1 cm apart, first one at 5 cm, double hash=10cm, triple hash=15 cm
- Should see 8-14 cm hash mark at skin entry site in normal size adult, read anesthesia note to check depth at insertion

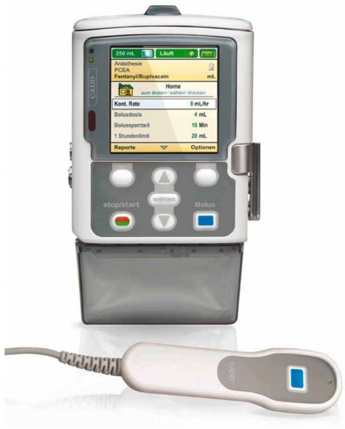

- Use the “silence” instead of “acknowledge” for repeat/ reminder alarms. Use "acknowledge" only when changing the bag.
- Recommendation: set reservoir volume for new bag at 40 ml below volume of bag to prevent bag running dry
- Assess orthostatic vital signs upon initial activity

# Epidural Analgesia

- May be ordered as continuous infusion with or without patient demand dose or programmed intermittent bolus (PIB). PIB provides higher pressure drug delivery during bolus which potentially provides increased spread and more effective anesthesia.
- Check Epidural is Secure

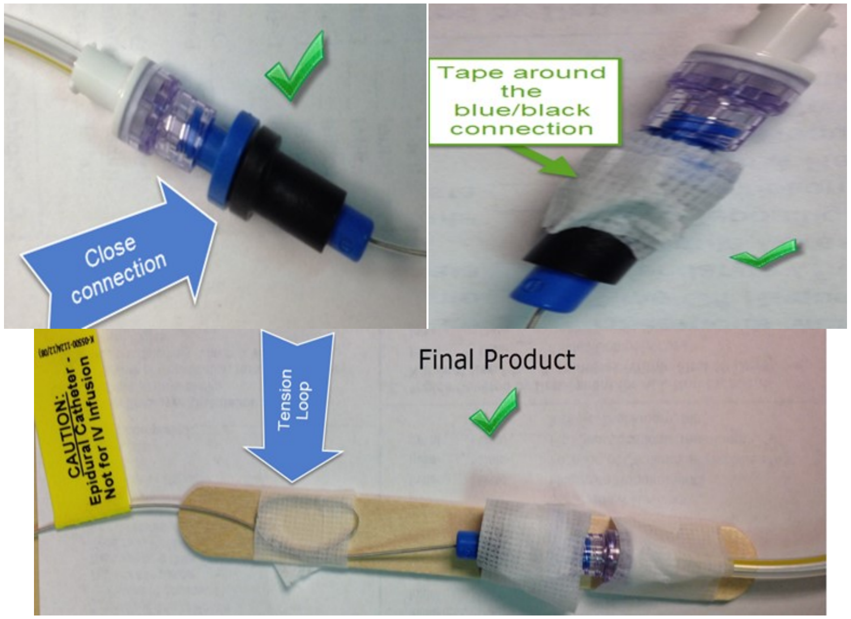


*If line falls out, then cover in sterile gauze & contact pain service. Reconnection should be an aseptic technique done by providers.*

- Epidural tubing NOT routinely changed with medication change, but consult pain service if concerns
  - Only PACU RNs, adult critical care RNs, and providers may attach epidural catheters to infusion tubing



# Multimodal Analgesia

- 2 or more analgesics target different pain mechanisms

**WHY?**

- Maximize overall pain relief by preventing analgesic gaps & needing lower doses of each of the drugs alone, particularly opioids, leading to less adverse effects

→ GREATER PAIN CONTROL

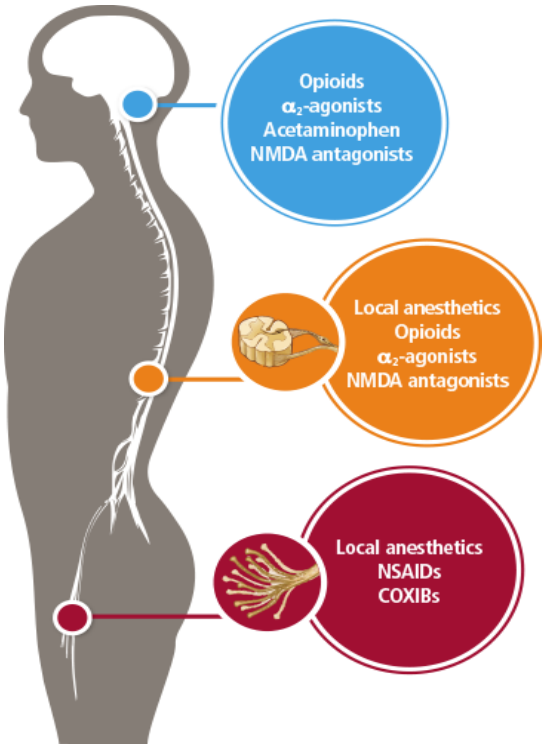




http://ofirmev.com/

# High Risk Medication Management for PCA, Epidural, Peripheral Nerve Infusion & Ketamine

- 2 RN independent check before start infusion, each order/ pump change, shift change, medication bag change
- Assess & document upon initiation of infusion, every 2 hours for first 12 hours, then every 4 hours for remainder infusion:
  - Pain intensity
  - Respiratory rate
  - Sedation
  - Oxygen saturation

Re-start the initial monitoring requirements for any drug change or dose increases

Highly recommend making yourself a checklist for every 2 hour mark & auditing yourself at end of your shift!



# Patient Controlled Analgesia

- Educate patient (and family) about use of button, safety measures of lockout time & hourly limit, and that **only the patient** may press the button
- Must be infused with continuous IV infusion
- Treat bolus doses like a PRN IV dose in terms of assessment and reassessment monitoring (within 30 min)

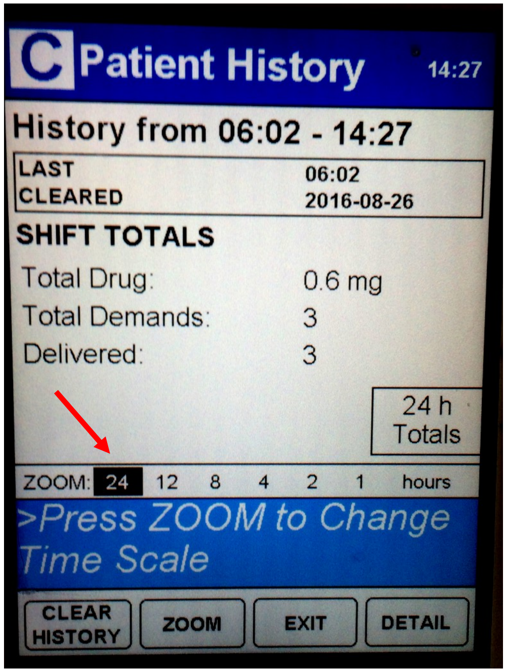


- Zoom to 24 hrs on Shift Totals screen to then clear and document **total drug** AND **volume infused** at end of 12 hour shift

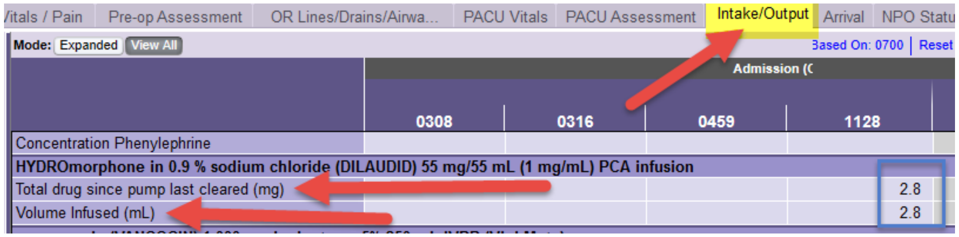

Supplement: Supplementary file 2 — Additional file 2. Nursing Analgesic Resources Booklet [file 13741_2020_153_MOESM2_ESM.pdf]
