# Supplementary material for: Using provider-focused education toolkits can aid enhanced recovery programs to further reduce patient exposure to opioids
Source: Perioper Med (Lond). 2020 Jul 9;9:21. doi: 10.1186/s13741-020-00153-5 (PMC7346381; doi:10.1186/s13741-020-00153-5)
Supplement: Supplementary file 3 — Additional file 3. Opioid conversion tables. [file 13741_2020_153_MOESM3_ESM.pdf]

Opioid	Route	Unconverted Dose	Dose Converted to mg (if needed)	Multiplication Factor to get IV ME	IV Morphine Equivalents	Multiplication Factor to get OME	Oral Morphine Equivalents
Hydrocodone	PO	1 mg	N/A	N/A	N/A	1	1mg
Morphine	IV	1 mg	N/A	N/A	N/A	3	3mg
Morphine	Epidural	1 mg	N/A	10	10mg	3	30mg
Morphine	Intrathecal	1mg	N/A	100	100mg	3	300mg
Morphine	PO	1 mg	N/A	N/A	N/A	1	1mg
Fentanyl	IV	1 mcg	0.001mg	100	0.1mg	3	0.3mg
fentanyl	Intrathecal	1mcg	0.001mg	1000	1mg	3	3mg
Fentanyl	Epidural	1 mcg	0.001mg	100	0.1mg	3	0.3mg
Hydromorphone	IV	1 mg	N/A	6.67	6.67mg	3	20mg
Hydromorphone	PO	1 mg	N/A	N/A	N/A	4	4mg
Hydromorphone	Intrathecal	1mg	N/A	166.75	166.75mg	3	500.25mg
Hydromorphone	Epidural	1 mg	N/A	5	5mg	3	15mg
Meperidine	IV	1 mg	N/A	0.1	0.1mg	3	0.3mg
Meperidine	PO	1 mg	N/A	N/A	N/A	0.1	0.1mg
Tramadol	PO	1 mg	N/A	N/A	N/A	0.25	0.25mg
Tramadol	IV	1 mg	N/A	0.1	0.1mg	3	0.3mg
Codeine	PO	1 mg	N/A	N/A	N/A	0.15	0.15mg
Codeine	IV	1 mg	N/A	0.1	0.1mg	3	0.3mg
Oxycodone	PO	1 mg	N/A	N/A	N/A	1.5	1.5mg
Oxycodone	IV	1 mg	N/A	1	1mg	3	3mg
Propoxyphene	PO	1 mg	N/A	N/A	N/A	0.15	0.15mg
Oxymorphone	PO	1 mg	N/A	N/A	N/A	3	3mg
Oxymorphone	IV	1 mg	N/A	10	10mg	3	30mg
Levorphanol	IV	1 mg	N/A	5	5mg	3	15mg
Levorphanol	PO	1 mg	N/A	N/A	N/A	7.5	7.5mg
Nalbuphine	IV	1 mg	N/A	1	1mg	3	3mg
Buprenorphine	IV	1mg	N/A	33.3	33.3mg	3	100mg
Fentanyl	Transdermal	1 mcg/h	0.001mg/h	N/A	N/A	3000	3mg/h
Buprenorphine	Transdermal	1 mcg/h	0.001mg/h	N/A	N/A	1500	1.5mg/h
Tapentadol	PO	1 mg	N/A	N/A	N/A	0.25	0.25mg
Pentazocine	IV	1 mg	N/A	0.33	0.33mg	3	0.99mg
